# Human multipotent hematopoietic progenitor cell expansion is neither supported in endothelial and endothelial/mesenchymal co-cultures nor in NSG mice

**DOI:** 10.1038/s41598-019-49221-x

**Published:** 2019-09-09

**Authors:** Stefan Radtke, André Görgens, Symone Vitoriano da Conceição Castro, Lambros Kordelas, Angela Köninger, Jan Dürig, Michael Möllmann, Peter A. Horn, Bernd Giebel

**Affiliations:** 1 Institute for Transfusion Medicine, University Hospital Essen, University of Duisburg-Essen, Essen, Germany; 2 Clinical Research Center, Department of Laboratory Medicine, Karolinska Institutet, Stockholm, Sweden; 3 CAPES Foundation, Ministry of Education of Brazil, Brasília, 70040-020 Brazil; 4 Department of Bone Marrow Transplantation, University Hospital, University Duisburg-Essen, Essen, Germany; 5 Department of Gynecology and Obstetrics, University Hospital Essen, University of Duisburg-Essen, Virchowstr. 179, D-45147 Essen, Germany; 6 Department of Hematology, University Hospital Essen, University of Duisburg-Essen, Essen, Germany

**Keywords:** Haematopoietic stem cells, Stem-cell niche

## Abstract

Endothelial and mesenchymal stromal cells (ECs/MSCs) are crucial components of hematopoietic bone marrow stem cell niches. Both cell types appear to be required to support the maintenance and expansion of multipotent hematopoietic cells, i.e. hematopoietic stem cells (HSCs) and multipotent progenitors (MPPs). With the aim to exploit niche cell properties for experimental and potential clinical applications, we analyzed the potential of primary ECs alone and in combination with MSCs to support the *ex vivo* expansion/maintenance of human hematopoietic stem and progenitor cells (HSPCs). Even though a massive expansion of total CD34^+^ HSPCs was observed, none of the tested culture conditions supported the expansion or maintenance of multipotent HSPCs. Instead, mainly lympho-myeloid primed progenitors (LMPPs) were expanded. Similarly, following transplantation into immunocompromised mice the percentage of multipotent HSPCs within the engrafted HSPC population was significantly decreased compared to the original graft. Consistent with the *in vitro* findings, a bias towards lympho-myeloid lineage potentials was observed. In our conditions, neither classical co-cultures of HSPCs with primary ECs or MSCs, even in combination, nor the xenograft environment in immunocompromised mice efficiently support the expansion of multipotent HSPCs. Instead, enhanced expansion and a consistent bias towards lympho-myeloid committed LMPPs were observed.

## Introduction

Self-renewal and differentiation of multipotent hematopoietic stem and progenitor cells (HSPCs) need to be highly controlled in order to warrant a life-long supply of mature blood cells. Loss, exhaustion, or acquisition of DNA damage in these HSPCs can lead to imbalanced lineage-output, anemia or hematological malignancies. The common treatment for many of these diseases involves the transplantation of allogeneic hematopoietic stem cells (HSCs) from HLA-matched unrelated or related donors^1^. However, matched donors can be difficult to identify for some patients and transplantation of partly-matched, unrelated donors can result in life-threatening side-effects such as Graft-vs-Host disease (GVHD)^2–5^.

To overcome these limitations, many researchers aim to use umbilical cord blood (UCB)-derived HSPCs for allogeneic stem cell transplantation (alloSCT), which have been shown to require a lower cell dose per kg body weight, and HLA-mismatches are better tolerated by the host^6,7^. Since the HSPC yield of a single UCB unit in many cases is insufficient to transplant adults, a variety of *ex vivo* cultures conditions supporting the expansion of multipotent HSPCs has been reported within the last years^8–12^. One promising strategy employs a feeder-based co-culture system to mimic the bone marrow (BM) stem cell niche for the expansion of multipotent HSPCs for experimental, pre-clinical as well as clinical approaches^13–16^, reviewed in^17,18^.

**Figure 1:**
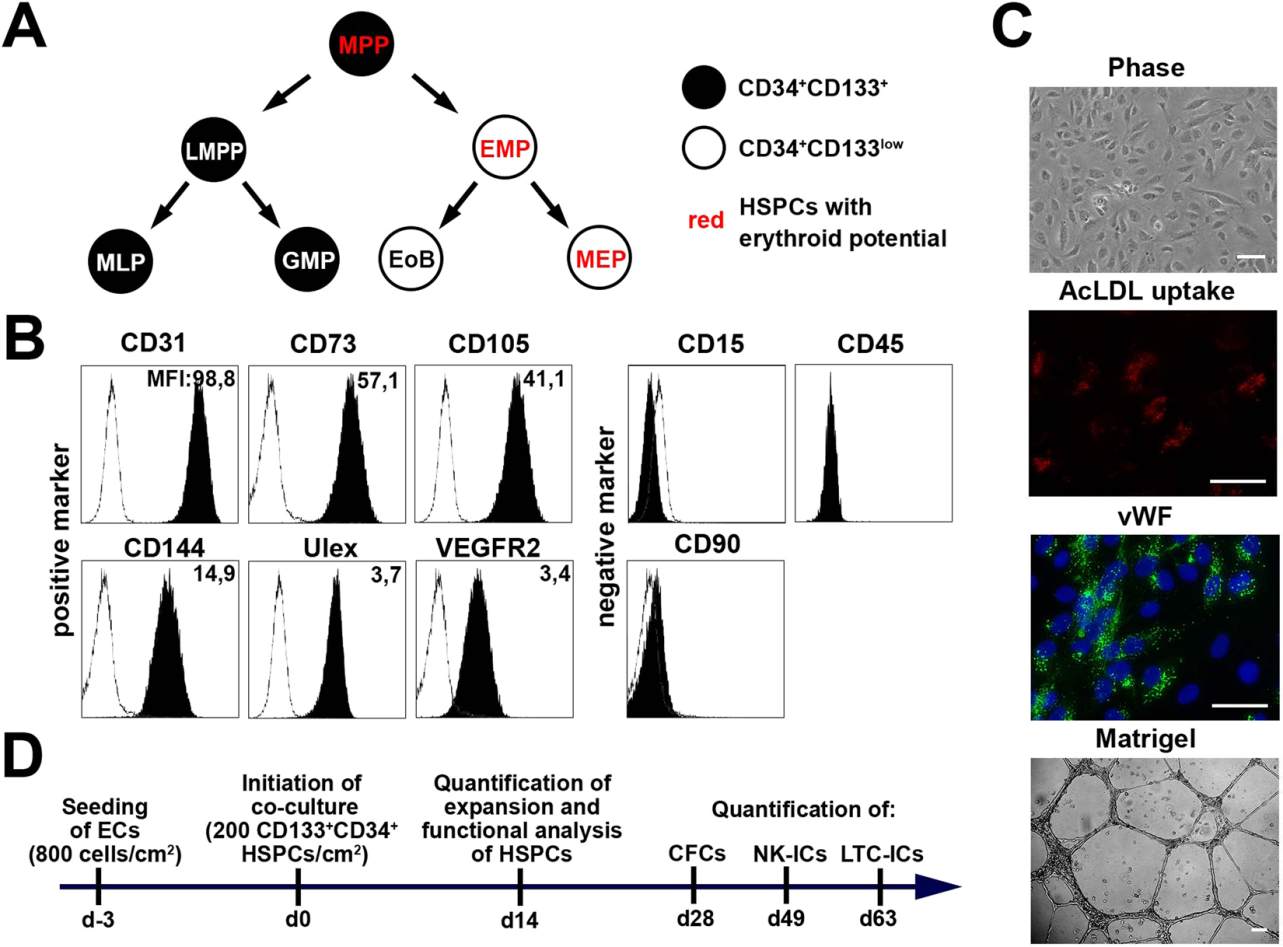
Experimental strategy and characterization of primary human ECs. **(A)** Revised model of the human hematopoiesis describing an early segregation of lympho-myeloid and erythro-myeloid lineages^19^. HSPCs with erythroid potentials are highlighted in red with HSCs and MPPs being the only CD133-expresing progenitors with erythroid capabilities. MLP: multi-lymphoid progenitor; GMP: granulocyte-macrophage progenitor; EoBP: eosinophil-basophil progenitor; MEP: megakaryocytic-erythroid progenitor. **(B)** Representative analyses of the cell surface marker expression on HUVEC 1 (black histograms) in comparison to isotype-controls (white histogram). Numbers indicate the mean fluorescence intensity (MFI) of the specific staining. **(C)** Representative morphology of ECs (phase contrast), uptake of AcLDL (red), intracellular storage of vWF in Weibel-Palade bodies (green) and formation of tube-like structures in the Matrigel assays of isolated ECs. (Scale-bars: 50 µm). **(D)** Experimental design for the co-culture of human ECs and HSPCs.

The quantification of multipotent HSPCs is commonly performed according to the lineage-relationships proposed by the classical model of human hematopoiesis. According to this classical model, HSCs and multipotent progenitors (MPPs) are the only cells containing both myeloid as well as lymphoid differentiation potentials. However, the classical model of hematopoiesis has meanwhile been challenged by several groups proposing alternative lineage-relationships and read-outs for multipotent HSCs/MPPs^19–22^. In this context, we have shown that human CD133^+^CD45RA^−^CD34^+^ HSPCs are enriched for multipotent HSPCs^19^. *In vitro*, more than 80% of these cells perform asymmetric cell divisions to create one CD133^+^CD45RA^+^CD34^+^ and one CD133^−^CD45RA^−^CD34^+^ daughter cell. While CD133^+^CD45RA^+^CD34^+^ cells inherit the potential to create all lymphocytes and neutrophils, CD133^−^CD45RA^−^CD34^+^ cells are limited to erythroid, megakaryocytic, basophilic and eosinophilic potentials. The first cell fulfills the criteria of LMPPs^23^ and the latter ones correspond to cells that are commonly known as common myeloid progenitors (CMP). As we demonstrated that they are not able to form neutrophils, it is no longer justified to interpret them as CMPs. Accordingly, we re-named them to erythro-myeloid progenitors (EMPs) and proposed a revised model for human hematopoiesis in which neutrophils arise from the same branch as lymphocytes (Fig. 1A)^24^. The segregation of lineage potentials as predicted in our revised model was recently confirmed in mouse^20^ as well as in human steady state BM hematopoiesis using single cell transcriptomics^25^. According to the proposed model, multipotent HSPCs can easily and objectively be identified as CD133 expressing HSPCs containing erythroid differentiation potentials (Fig. 1A)^26^.

Using this definition as read-out to identify multipotent human HSPCs following *in vitro* expansion, we recently re-evaluated the reported potential of murine stromal cell lines (AFT024, OP9, MS5) as well as human mesenchymal stromal cell (MSCs) from various tissues to support the *ex vivo* expansion of UCB-derived HSCs/MPPs^15^. In these experiments, none of the tested culture conditions supported the expansion or maintenance of primitive CD133^+^ HSPCs with erythroid differentiation potentials. However, all tested conditions demonstrated robust expansion of phenotypical and functional LMPPs.

While these experiments were exclusively performed with a mono-layer of murine stromal cells or human MSCs, the cellular composition of the BM stem cell niche is known to be much more complex and involves a variety of different cell types, signaling molecules as well as other soluble/cell-bound factors^27–31^. Another crucial cellular component of the stem cell niche and being a major contributor to HSC maintenance has recently been attributed to endothelial cells (ECs)^32,33^. Synergistically with MSCs, both cell types were shown to be essential components for HSC maintenance, and knockout of either cell type led to specific depletion of phenotypically and functionally distinct HSC/MPP subsets^32,33^.

Based on these findings, we decided to investigate whether primary ECs either alone or in combination with MSCs support the *ex vivo* expansion and/or maintenance of CD133^+^ HSPCs with erythroid differentiation potential. Furthermore, we tested the expansion capabilities of HSCs/MPPs in an *in vivo* environment, i.e. in a xenograft repopulation model in immunodeficient NSG (Non-obese diabetic scid gamma) mice.

## Results

### Primary ECFCs and HUVECs are phenotypically and functionally homogeneous

Human ECs can be easily generated from various tissues. Here, we raised ECs from five independent UCB units termed endothelial colony forming cells (ECFCs) and from umbilical veins of five different umbilical cords, classically termed human umbilical vein endothelial cells (HUVECs). Within our analyses, we did not detect any striking phenotypic differences between ECFCs and HUVECs. All ECs homogenously expressed the cell surface markers CD31, CD73, CD105, CD144, VEGFR2 and bound the lectin Ulex (Figs 1B, S1). Expression of hematopoietic (CD15 and CD45) and mesenchymal (CD90) cell surface markers was not detected (Figs 1B, S1)^34^. ECs were able to take up acetylated low-density lipoprotein (AcLDL), to store Von Willebrand Factor (vWF) in Weibel-Palade bodies and to form tube-like structures in Matrigel assays (Figs 1C, S2)^34^. In summary, all obtained primary ECFCs and HUVECs fulfilled the widely-accepted criteria of bona fide ECs.

### ECFCs and HUVECs promote expansion of CD133^+^CD34^+^ HSPCs

**Figure 2:**
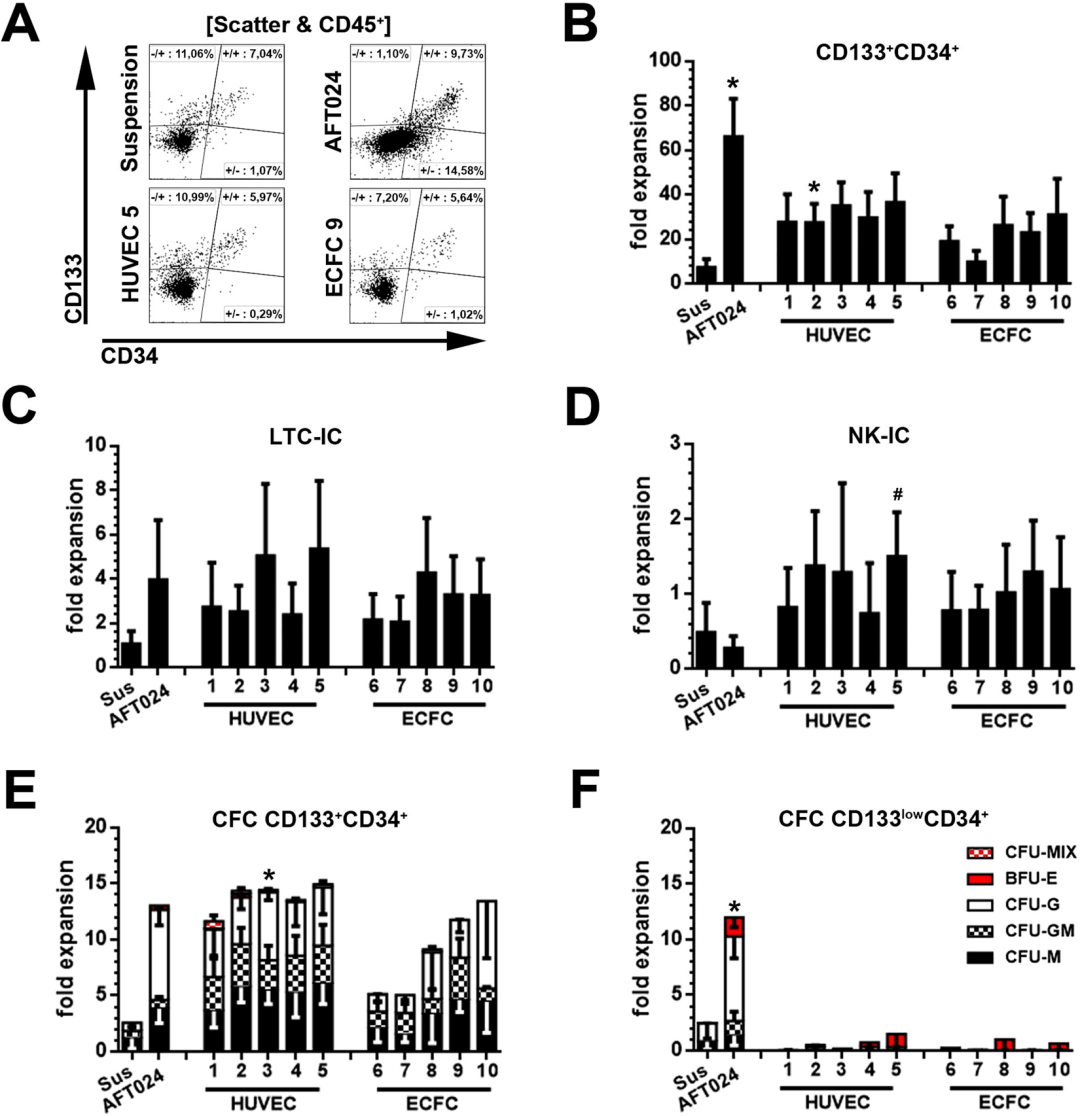
Phenotypical and functional characterization of CD133^+^CD34^+^ cells expanded in co-culture with primary ECs. **(A)** Representative gating strategy for the quantification of phenotypical CD133^+^CD34^+^ and CD133^low^CD34^+^ HSPCs after 14 days of co-culture. Fold-expansion of **(B)** CD133^+^CD34^+^ cells revealing (n = 4 for HUVEC 3, all other n = 5) **(C)** LTC-IC (n = 4 for HUVEC 1 + 3 + 5, all other n = 5), **(D)** NK-IC (n = 3 for Sus, HUVEC 3 and ECFC 7, all other n = 4) and **(E)** CFC potentials (CD133^+^: n = 3 for ECFC 1, all other n = 4; CD133^low^: n = 3 for all) in co-culture with human ECs. **(F)** CFC potential of CD133^low^CD34^+^ cells derived from corresponding co-cultures. Co-cultures with AFT024 stromal cells (AFT024) and suspension cultures (Sus) were used as controls. Primitive hematopoietic cells containing CFC potentials were subdivided based on colony type (BFU-E, CFU-M, CFU-G and CFU-GM and CFU-MIX) (x-axis: independent experiments with different donor-derived HUVECs or ECFCs, respectively; statistics: mean ± SEM; *p-value < 0.05, tested against Sus; ^#^p-value < 0.05, tested against AFT024).

To test the hematopoietic support of ECFCs and HUVECs, ECs were co-cultured for two weeks with sort-purified UCB-derived CD133^+^CD34^+^ cells as previously reported (Figs 1D, S3)^15^. Suspension cultures and co-cultures with the murine stromal cells AFT024 were used as controls. At the end of co-culture, cells were harvested, the composition of hematopoietic progeny was analyzed by flow-cytometry, and the expansion of phenotypical subset quantified (Figs 2, S4A).

A robust expansion of CD45^+^ hematopoietic cells lacking expression of CD34 and CD133 was observed in all conditions (Figs 2A, S4A). Despite significant loss of CD34 and CD133 expression, the total number of CD133^+^CD34^+^ cells increased in all conditions. The average fold-expansion of initially seeded CD133^+^CD34^+^ HSPCs in co-culture with ECs ranged from 9.8 ± 4.7-fold (ECFC 7) to 36.5 ± 13.0-fold (HUVEC 5) and was higher than in the suspension cultures (7.3 ± 3.7-fold), but lower than in the AFT024 (66.0 ± 17.1-fold) co-cultures (Fig. 2B). In conclusion, isolated human ECFCs and HUVECs support the expansion of phenotypically-primitive CD133^+^CD34^+^ HSPCs.

### Lympho-myeloid bias of culture-expanded CD133^+^CD34^+^ HSPCs

The phenotype of culture-expanded human HSPCs has previously been shown to not necessarily represent the functional properties observed in freshly isolated subsets^35,36^. Consequently, culture-derived CD133^+^CD34^+^ and CD133^low^CD34^+^ cells were sort-purified and transferred into *in vitro* read-outs to determine the expansion of functionally primitive cells. Progenies were analyzed for their erythro-myeloid (colony-forming cell; CFC), long-term myeloid (long-term culture initiating cells; LTC-IC) and long-term lymphoid (NK cell initiating cell; NK-IC) potentials (Fig. 2).

All ECs supported the expansion of LTC-ICs with an average expansion rate ranging from 2.0 ± 1.1-fold (ECFC 7) to 5.0 ± 2.9-fold (HUVEC 5). In suspension cultures, cells revealing LTC-IC potentials did not expand (1.07 ± 0.2-fold), while expansion of cells with LTC-IC potentials was slightly higher on AFT024 (3.95 ± 2.68-fold) than in comparison to most of the EC co-cultures (Fig. 2C). Little expansion (>1-fold; <2-fold) of NK-ICs was supported in co-culture with HUVEC 2, 3 and 5 as well as ECFC 8, 9 and 10, whereas NK-ICs were lost (<1-fold) in suspension culture as well as in co-culture with the murine stromal cells AFT024 or the HUVEC 1 and 4 and the ECFC 6 and 7 (Fig. 2D). CD133^+^CD34^+^ HSPCs containing CFC potential were expanded under all conditions tested, including suspension cultures and AFT024 co-cultures ranging from 3.8 ± 2.3-fold (ECFC6) to 14.4 ± 2.6-fold (HUVEC3, Fig. 2E). Obtained CD133^+^CD34^+^ progenitors predominantly revealed granulocyte (CFU-G), monocyte (CFU-M) or granulocyte/monocyte (CFU-GM) potentials and nearly entire absence of erythroid progenitors. CD133^low^CD34^+^ HSPCs revealing CFC-potentials were hardly detected in co-cultures with human ECs and restricted to erythro-myeloid lineages (Fig. 2F).

Thus, our data imply that neither MSCs^15^ nor ECs alone are sufficient to promote expansion or maintenance of CD133^+^ HSPCs with lymphoid, myeloid as well as erythroid differentiation potential.

### Combination of EC and MSC stroma improves maintenance of lymphoid potentials

**Figure 3:**
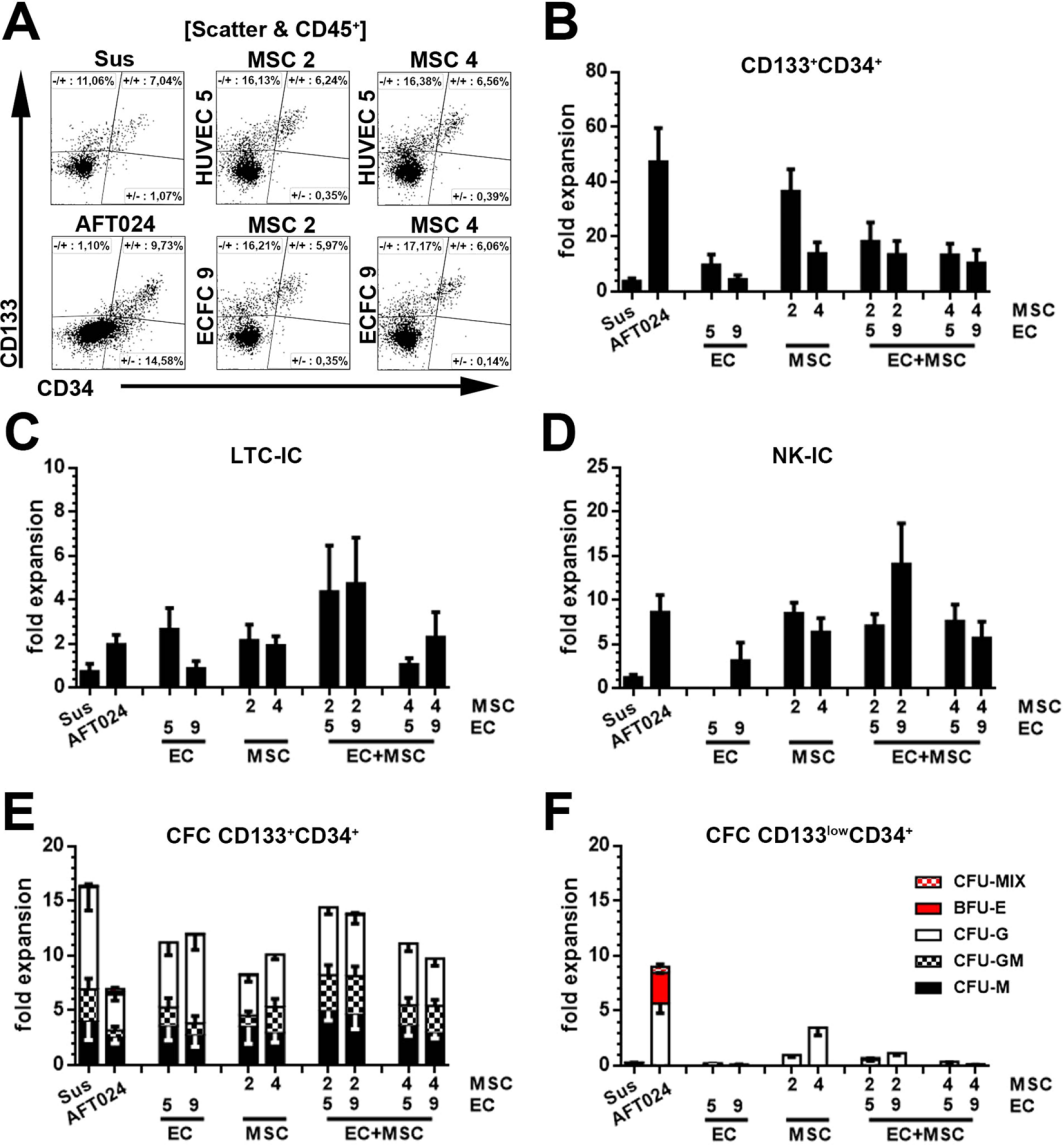
Expansion and functional characterization of CD133^+^CD34^+^ cells in co-culture with primary human ECs and MSCs. **(A)** Flow-cytometric assessment of hematopoietic progeny (CD45^+^) cells after expansion in co-culture with primary human ECs (EC5 = HUVEC 5 and EC9 = ECFC 9) and MSCs (MSC2 = BM MNC 2 and MSC4 = BM Fat 4 as described in Radtke *et al*. 2016^15^). Fold-expansion of **(B)** phenotypically CD133^+^CD34^+^ HSPCs revealing (n = 5) **(C)** LTC-ICs (n = 4 for EC2 + MSC2 and EC5 + MSC4, all other n = 5), **(D)** NK-ICs (n = 3 for EC9 + MSC2, all other n = 5) and **(E)** CFC potentials (n = 5). **(F)** CFC potential of CD133^low^CD34^+^ cells derived from corresponding co-cultures. Primitive hematopoietic cells revealing CFC potentials were subdivided regarding their arising colony types (BFU-E, CFU-M, CFU-G and CFU-GM and CFU-MIX) (x-axis: independent experiments with different donor-derived HUVECs or ECFCs, respectively; statistics: mean ± SEM; *p-value < 0.05, tested against Sus; ^#^p-value < 0.05, tested against AFT024).

ECs and MSCs have both been reported to be essential components of the BM stem cell niche^32,37^. Therefore, we decided to test HSPC expansion in triple cultures with MSCs and ECs. Initially, we searched for culture conditions being permissive for all cell types. MSCs, which had been established before^15^, and ECs remained viable in basal HSPC expansion media (IMDM supplemented with 20% FBS and the cytokines SCF, TPO and FLT3L) if FGF and EGF were added. Using these conditions, we confirmed that after 14 days of co-culture MSCs and ECs were still present and formed a homogenous monolayer with approximately equal MSC and EC cell numbers **(**Figs S4B and S4C). Although HSPCs expanded quite well in these cultures, apparently the addition of FGF and EGF reduced their overall expansion rates which were much higher in our previous experiments in which we expanded HSPCs on MSCs in HSPC expansion media not being supplemented with FGF and EGF^15^ (data not shown). Similar to previous co-culture experiments, culture-expanded hematopoietic progeny were comprehensively analyzed by flow-cytometry and introduced into functional read-outs (Figs 3, S4).

Upon analyzing the triple cultures at culture day 14, most expanded hematopoietic cells belonged to the group of CD45^+^CD133^−^CD34^−^ cells (Figs 3A, S4D); still, expansion of CD34^+^ HSPCs was recorded. The average fold-expansion of CD133^+^CD34^+^ HSPCs in control co-cultures was in the range from 4.4 ± 0.68-fold (EC9) to 36.6 ± 3.5-fold (MSC2). In triple cultures, the expansion rate was between 10.5 ± 2.1-fold (MSC4/EC9) and 18.4 ± 3.0-fold (MSC2/EC5) (Fig. 3B).

Functional analyses showed that CD133^+^CD34^+^ LTC-ICs as well as NK-ICs were maintained or expanded under all conditions tested, with the greatest expansion in the triple cultures containing MSC2 and EC9 stromal cells (LTC-IC: 4.7 ± 0.9-fold; NK-IC: 14.1 ± 2.0-fold) (Fig. 3C,D). CD133^+^CD34^+^ HSPCs demonstrating CFC potentials were also expanded under all conditions with the lowest fold expansion on MSC4 (3.1 ± 1.4-fold) and the highest in triple cultures with MSC2 and EC5 (11.2 ± 7.6-fold) (Fig. 3E). However, no CD133^+^CD34^+^ cells with erythroid potentials were detected in any of the tested conditions (Fig. 3E). CFC frequencies in obtained CD133^low^CD34^+^ fractions were generally relative low, and all obtained colonies were classified as CFU-G colonies (Fig. 3F).

In summary, the combination of ECs and MSCs as feeder layer for HSPC cultures resulted in expansion of HSPCs with lympho-myeloid potentials but did not support the expansion or maintenance of multipotent HSPCs, defined as CD133^+^CD34^+^ cells with erythroid potentials.

Our data imply that neither MSC/EC co-cultures alone nor the combination of both support the *ex vivo* expansion or maintenance of multipotent HSPCs. Hypothesizing that other crucial cell types or factors present in the HSC niche are missing. Due to the observed effect of EGF and FGF in our triple cultures as well as the potential impact of serum on the self-renewing potential of multipotent HSPCs, we decided to study human HSPCs kinetics in an *in vivo* environment.

### The xenogeneic environment in NSG mice promotes expansion of human lympho-myeloid progenitors but not of multipotent HSPCs

**Figure 4:**
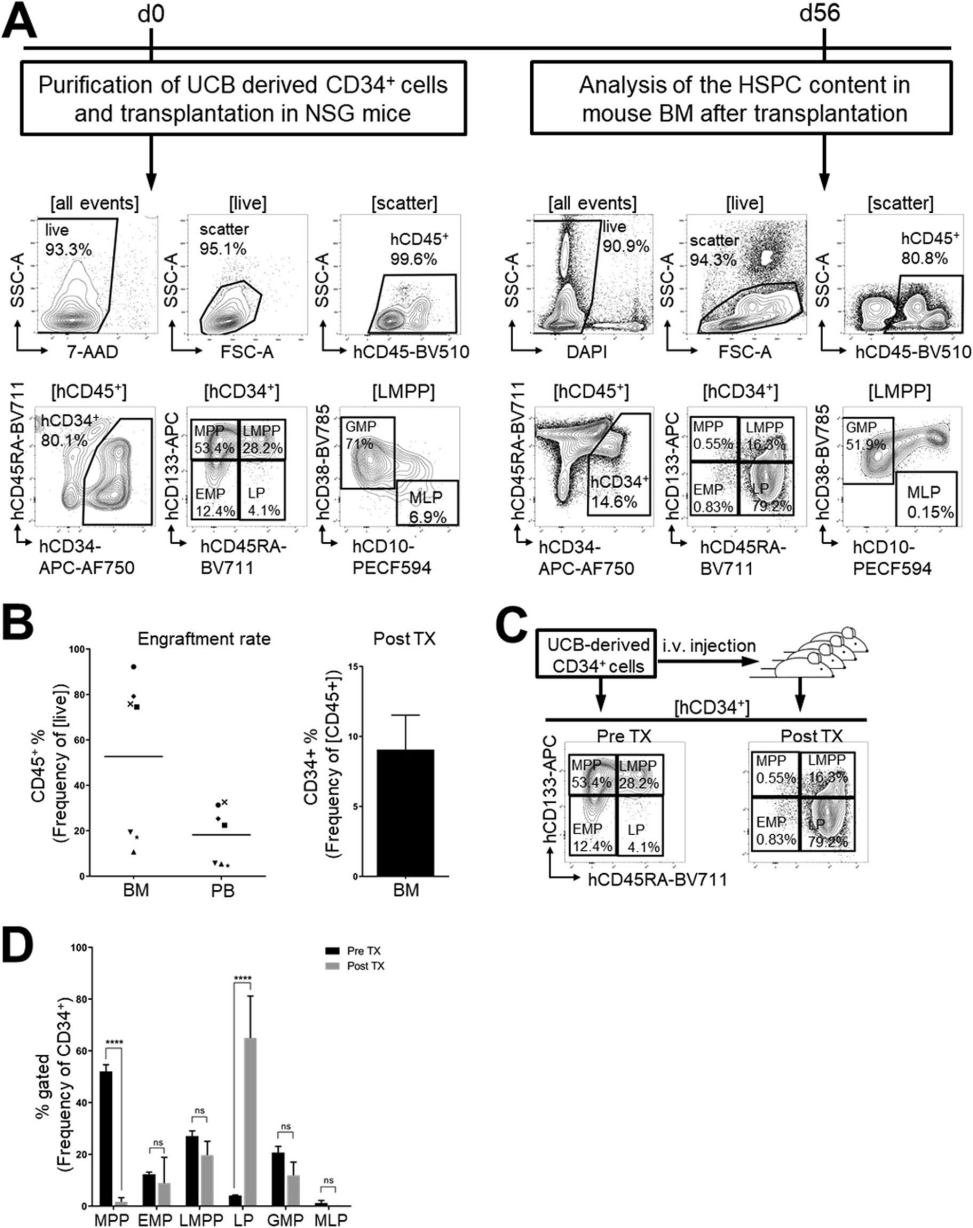
HSPC repopulation assay in immunodeficient mice. (**A)** Experimental design and gating strategy. UCB-derived CD34^+^ cells were purified, phenotypically characterized by flow cytometry and transplanted into NSG mice. Murine BM was harvested 8 weeks post-transplant (TX) and analyzed for HSPC content. **(B)** Bar graphs reflect human CD45^+^ cell engraftment in the BM and PB as well as frequency of CD34^+^ cells in the BM of transplanted mice. **(C)** Representative flow cytometric assessment of phenotypically defined HSPC subsets in freshly-isolated UCB-derived CD34^+^ cells and engrafted human CD34^+^ cells 8 weeks post-transplant. **(D)** Quantitative comparison of HSPC subsets pre- and post-transplantation. Bar graphs: Mean ± SD. Statistical analysis: Two-way ANOVA (n = 7).

For the *in vivo* studies, we have chosen the NSG mouse xenograft model which has been used in several studies to demonstrate human “HSC expansion” before^38–43^. Experimentally, we analyzed freshly isolated UCB-derived CD34^+^ cells with an enhanced marker panel^24,44^, transplanted these cells into sub-lethally irradiated NSG mice, and analyzed the phenotypical composition of engrafted human HSPCs 8 weeks post-transplant (Fig. 4A).

UCB-derived CD34^+^ cells were sub-classified based on their CD133 and CD45RA expression as MPPs (CD34^+^CD133^+^CD45RA^-^), as lympho-myeloid progenitors (CD34^+^CD133^+^CD45RA^+^), as EMPs (CD34^+^CD133^low/−^CD45RA^−^) or late progenitors (LPs: CD34^+^CD133^low/−^CD45RA^+^). Lympho-myeloid progenitors were sub-categorized by their CD10 and CD38 expression as LMPPs (CD34^+^CD133^+^CD45RA^+^CD38^low^CD10^−^), granulocyte-macrophage progenitors (GMPs: CD34^+^CD133^+^CD45RA^+^CD38^+^CD10^−^), and mixed-lymphoid progenitors (MLPs: CD34^+^CD133^+^CD45RA^+^CD38^−^CD10^+^) (Fig. 4A). In agreement to our previous reports^19,24,44^, the frequency of each progenitor subset within the transplanted CD34^+^ cell preparations (before transplantation) was 52.1 ± 2.6% MPPs, 12.3 ± 0.8% EMPs, 27.1 ± 2.1% LMPPs, 4.1 ± 0.2% LPs, 20.7 ± 2.4% GMPs and 1.2 ± 1% MLPs (Fig. 4A).

Eight weeks’ post transplantation, the BM of recipient mice were comprehensively analyzed via flow cytometry (Fig. 4A). Both BM and PB showed engraftment of human cells at a rate of 52.7 ± 35.1% and 18.2 ± 12.6%, respectively. 9.1 ± 6.5% of BM-resident human cells expressed CD34 (Fig. 4B). Detailed phenotypical analysis of human CD34^+^ cells in the murine BM revealed significantly lower frequencies of MPPs (1.6 ± 1.5%) as well as increased frequencies of LPs (65 ± 16.2%) in comparison to transplanted UCB-derived CD34^+^ cell fractions. Furthermore, we noticed a trend for all other more primitive HSPC subsets to be less frequent within the human HSPC fraction isolated from the murine BM when compared to frequencies in fresh UCB derived CD34^+^ cells: 8.9 ± 10% EMPs, 19.8 ± 5.3% LMPPs, 11.9 ± 5.1% GMPs and no MLPs (Fig. 4C,D).

In conclusion, the *in vivo* data imply that like *in vitro*, multipotent human HSPCs are not maintained or significantly expanded in the xenogeneic BM environment of NSG mice, which are used as a gold-standard *in vivo* xenograft transplantation model.

## Discussion

Here, we systematically evaluated ECs as well as the combination of ECs and MSCs for the *ex vivo* expansion of human HSCs. None of the *ex vivo* culture conditions tested promoted the expansion or sustained maintenance of human UCB-derived multipotent human HSPCs. Instead, all conditions efficiently promoted the expansion of lympho-myeloid restricted progenitors with LTC-IC and NK-IC potential. Similarly, upon transplantation of freshly-isolated human UCB-derived CD34^+^ cells into NSG mice we observed specific loss of HSCs/MPPs and a significant accumulation of LMPPs. From these data we conclude that both, the *in vitro* as well as the *in vivo* conditions used in our experiments are not permissive to allow expansion of multipotent HSPCs.

As *in vitro* and xenogeneic conditions will never be physiological, we cannot exclude that other experimental conditions may affect the HSPC biology differently and promote multipotent HSPC maintenance or even expansion. However, comparable to the results of the NSG mouse study presented here, and as discussed in more detail below, we and others observed a strong decline in multipotent HSPC frequencies following allogeneic and autologous HSPC transplantation into immunocompromised patients^45^ (Kordelas *et al*., #BMT-2019-466R)^46^. Thus, the self-renewal capability of multipotent HSPCs might be markedly lower than generally assumed. Instead of self-renewal, the *in vitro* and *in vivo* environment tested here rather promoted the formation and expansion of HSPCs with lympho-myeloid differentiation capabilities.

Our findings seem to be contradictory to various studies reporting successful and efficient expansion of primitive human HSPCs in co-culture systems with primary ECs^41,47–49^ as well as more recently published conditions with genetically-modified ECs and MSCs^11,50^. Analyzing culture-expanded progeny with state-of-the-art *ex vivo* read-outs (CFC, LTC-IC, NK-IC assays etc.) and/or transplanting hematopoietic progeny into the mouse xenograft model to quantify the expansion of NOD/SCID repopulating cells (SRCs), all of the publications above demonstrate robust expansion of human HSPCs with lymphoid as well as myeloid differentiation/engraftment potential. As discussed in various publications^15,26,51^, the demonstration that neutrophils arise from the same branch as lymphocytes, and due to the fact that most papers screened in reconstituted immunocompromised mice for granulocytes and lymphocytes only to consider multipotency of engrafted cells, the interpretation of such analyses need to be carefully revisited. Indeed, we and others have demonstrated that LMPPs contain SCR activities being able to create neutrophils and lymphocytes but none of the EMP derivatives^19,23^. While until recently and according to the classical model of hematopoiesis the assessment of erythroid and megakaryocytic potentials was not required to demonstrate multipotency of human HSPCs, it is evident, that the read-out of individual differentiation potentials *in vitro* or *in vivo* in the mouse xenograft model as it had been performed until now, is not sufficient to confirm multipotency of transplanted HSPCs^26,51^. Unfortunately, the most frequently used mouse models do not efficiently support the long-term development of all human blood lineage^52–55^ and demonstrate only short-term support for the development and maturation of erythrocytes and megakaryocytes^56^. Furthermore, engraftment of lympho-myeloid primed human and baboon HSPCs lacking erythro-megakaryocytic potentials^19,51^, serial engraftment potential of myelo-megakaryocytic restricted progenitor cells^57^, as well as efficient and high-level short-term engraftment of multipotent progenitor cells^58,59^ challenge the mouse xenograft model as a unique read-out for HSCs/MPPs.

Current attempts to provide a reliable read-out for the multilineage differentiation potential of either freshly-isolated, gene-modified, or culture-expanded human HSPCs include further humanization of already existing mouse models^60–63^ or subcutaneous transplantation of *ex vivo* generated human ossicles to model a humanized BM niche in the mouse xenograft^64–66^. Both strategies were shown to improve human cell engraftment as well as more realistic lineage output similar to autologous transplantation. In addition, presence of a more human-like bone marrow environment enabled successful transplantation and engraftment of malignant blood cells, a read out that was not supported by conventional mouse xenograft models. To our best knowledge, assessment of human HSPC engraftment in these novel mouse models according to our proposed read-out of human HSCs/MPPs has not been performed, yet. Thus, it remains to be investigated whether HSC/MPP-enriched CD34^+^CD133^+^CD454RA^−^ cells with erythroid differentiation potential are maintained under any of these conditions. However, related to the results of our mouse experiments, upon comparing the frequencies of more primitive and more mature HSPC populations in human HSC-transplants and in the BM of successfully engrafted patients following alloSCT, a comparable shift of more primitive HSPC fates were observed in pediatric patients one year after alloSCT^45^ as well as in a cohort of adult patients four weeks following alloSCT using G-CSF mobilized HSC grafts (Kordelas *et al*., submitted)^46^. It might be argued that one year post transplant is too short to restore HPSC homeostasis in the BM of alloSCT patients. However, some early studies which investigated LTC-IC potentials of HSPCs of reconstituted BM up to 20 years following allogeneic BM transplantation (alloBMT) demonstrated that LTC-IC frequencies were much lower in successfully engrafted BM than in that of healthy donors and remained low over the years; still normal peripheral blood cell counts where recorded^67,68^. Since only MPPs and LMPPs contain LTC-IC potentials^19^, this data points towards the same direction as the data reported here. Together, these results imply that the self-renewal potential of human HSCs/MPPs is much lower than generally assumed. However, due to the lack of detailed information about the dynamics of the HSPC reconstitution in humans over time, it remains unknown whether the BM of conditioned human/mice is impaired in its ability to control and balance the composition of HSPCs or whether multipotent HSPCs have indeed reduced self-renewal capabilities.

In summary, *ex vivo* expansion and mouse xenograft engraftment of human HSCPs are consistently biased towards lympho-myeloid lineages. It will be interesting to test whether recently proposed culture media containing novel cytokines/cytokine-combinations, small molecules or advanced/genetically-modified stromal cells can prevent the lympho-myeloid shift and support the maintenance of phenotypical and functional HSCs/MPPs.

## Methods

### Cell sources

Human umbilical cord and UCB samples were obtained after informed consent according to the Declaration of Helsinki stating that the protocols used in this study have been approved by a local ethics committee/institutional review board of the clinical division at the University of Duisburg/Essen (Ethik-Kommission der medizinischen Fakultät der Universität Duisburg/Essen). Mononuclear cells (MNCs) from UCB were isolated by Ficoll (Biocoll Separating Solution, Biochrom AG, Berlin, Germany) density gradient centrifugation as described previously^69^.

### Hematopoietic cells

CD34^+^ cells were enriched from UCB MNCs by magnetic cell separation using the MidiMacs technique according to the manufacturer’s instructions (Miltenyi Biotec, Bergisch Gladbach, Germany).

### Mesenchymal stromal cells (MSCs)

Primary human MSCs were raised from either BM-derived MNCs or fat and cultured as described previously^15^.

### Endothelial stromal cells (ECs)

Primary human ECFCs were raised from UCB-derived MNCs and cultured as described previously^34^.

### Human umbilical vein endothelial cells (HUVECs)

Umbilical vein was purged twice with PBS to remove residual blood cells, clamped on one end, filled with 0.25% Trypsin and incubated for 5 min at 37 °C. Obtained cells were seeded in tissue culture 6-wells, non-adherent cells removed after 24 hours and adherent cells cultured as described previously^34^.

### Co-culture experiments

Sort-purified CD133^+^CD34^+^ cells (200/cm^2^) were seeded on stromal cells and co-cultured for 14 days (Fig. 1D). Co-culture was performed in IMDM (Lonza, Basel, Switzerland) supplemented with 20% FBS (Biochrom, Berlin, Germany), 100 U/ml penicillin, 100 U/ml streptomycin (Life Technologies, Karlsruhe, Germany) and SCF, TPO, FLT3-L (Peprotech, Rocky Hill, USA) each at 10 ng/ml final concentration with 50% of exchange of culture medium on day 7. For the co-culture experiments with HUVECs and ECFCs, the basic culture medium was supplemented with additional cytokines (FGF, EGF) from the endothelial growth medium (EGM, Lonza) to support survival and proliferation of endothelial cells throughout the entire 14 days of co-culture.

### Triple-culture experiments

Human MSCs and ECs were seeded at a ratio of 1:10 and confluency of 20–30%. Stromal cells were cultured over-night in HSPC expansion medium before sort-purified CD133^+^CD34^+^ cells (200/cm^2^) were added. For the co-culture of MSCs, ECs and human HSPCs, concentration of FGF and EGF were adjusted to 10 ng/ml in order to prevent excessive overgrowth and detachment of MSCs.

### Flow cytometric analysis and sorting

For flow-cytometric analysis hematopoietic cells and ECs were stained with different combinations of monoclonal fluorochrome-conjugated antibodies (see Supplemental Table 5) for at least 20 min at 4 °C. Propidium-Iodide (PI) or 7-Aminoactinomycin D (7-AAD) were used for dead cell exclusion. Appropriate isotype-matched, control monoclonal antibodies were used to determine the level of background staining in all experiments. Flow cytometric analyses were performed on a FC500 flow cytometer equipped with the CXP 2.2 software (Beckman Coulter) (Fig. 1C). Cells were sorted using a FACSAria I cell sorter. The sort-purity was routinely assessed by recovery of sorted cells and was >99.5%.

Samples from murine BM were stained with the following fluorochrome conjugated antibodies: anti-CD34-APC-AF750 (Beckman Coulter), anti-CD133-APC (Miltenyi Biotec), anti-CD45RA-BV711 (BioLegend), anti-CD38-BV785 (BD Biosciences), anti-CD10-PeCF594 (BD Biosciences) and CD45-BV510 (BD Biosciences). DAPI staining was used for excluding dead cells. All measurements were performed in a BD FACSAria^TM^III (Beckman Coulter) flow cytometer.

### Hematopoietic cell assays

For CFC assays, 400 sort-purified CD133^+^CD34^+^ or CD133^low^CD34^+^ cells were transferred into 1 ml MethoCult H4434 (StemCell Technologies, Vancouver, Canada). Hematopoietic colonies were scored after 14 days discriminating colony forming unit- (CFU-) macrophage (M), granulocyte (G), granulocyte-macrophage (GM) and burst forming unit-erythrocyte (BFU-E). Colonies consisting of erythroid and myeloid cells were scored as CFU-MIX. For LTC-IC and NK-IC assays 6000 CD133^+^CD34^+^ were sorted and analyzed in limiting dilutions as described previously^19^.

### NK cell initiating-cell (NK-IC) assay

Transferred cells were cocultured with the murine cell line AFT024 in Dulbecco modified Eagle medium (DMEM) and Ham F12-medium (Invitrogen) mixed in a 2:1 (vol/vol) relation containing 20% heat-inactivated human AB serum (Cambrex, Taufkirchen, Germany), ascorbic acid (20 mg/mL; Invitrogen), selenium selenite (50 µM; Invitrogen), 2-mercaptoethanol (25 µM), and ethanolamine (50 µM; Invitrogen). The following cytokines were added to these cultures: rhIL-2 (1000 U/mL), rhIL-3 (5 ng/mL), rhIL-7 (20 ng/mL), rhSCF (10 ng/mL), and rhFlt3L (10 ng/mL). At weekly intervals, half-media exchanges were performed using 10% instead of 20% human AB serum. Starting at week 2, the only cytokine added to the cultures was rhIL-2. After 5 to 7 weeks of culture, wells containing viable cells were harvested and cells were analyzed flow cytometrically using antibodies recognizing the NK cell-specific antigens CD16 and CD56 as well as CD3.

### Long-term culture initiating-cell (LTC-IC) assay

Transferred cells were cocultured with the murine cell line AFT024 in Iscove modified Dulbecco medium (IMDM; Invitrogen) supplemented with 12.5% fetal calf serum (FCS), 12.5% horse serum (Cell Systems), 2 mM L-glutamine (Invitrogen), 1000 U/mL penicillin, 100 U/mL streptomycin (Invitrogen), and 10^−6^ M hydrocortisone. Cultures were maintained for 5 weeks in a humidified atmosphere at 37 °C and 5% CO_2_ and fed once a week. At week 5 all wells were overlaid with clonogenic methylcellulose medium (Methylcellulose [Fluka, Buchs, Switzerland] in a final concentration of 1.12% containing IMDM and supplemented with 30% FCS, 3 U/mL erythropoietin [Cell Systems], and supernatant of the bladder carcinoma cell line 5637 [10%]). Wells were scored for the occurrence of secondary CFCs after an additional 2 weeks.

### EC assays

The angiogenic potential, the ability to uptake acetylated low-density lipoprotein (AcLDL) and the storage of von Willebrand Faktor (vWF) in Weibel-Palade bodies of ECFCs and HUVECs was analyzed as described previously^34^.

### Matrigel assay

The angiogenic potential of ECs was analyzed within the Matrigel assay (BD; Basement Membrane Matrix). Briefly, 40.000 ECs resolved in EGM-2 medium (Lonza) were transferred on top of 100 ml Matrigel that had previously been pipetted into 96-well plates and incubated at 37 °C for 30 min. After 15 h at 37 °C, images of individual wells were taken using an Axio Observer Z1 microscope platform, with either Plan-Neofluar x5 or x10 objective lenses (Zeiss).

### AcLDL-uptake

Regularly, 1 × 10^5^ ECs were fed with 10 mg/ml labelled acetylated low-density lipoprotein (DiI-AcLDL; Invitrogen, Darmstadt, Germany). Before the LDL uptake was analyzed after 1 h at 37 °C and 5% CO_2_ atmosphere, the supernatant was removed and the cells were washed twice using fresh EGM-2 medium.

### Von Willebrand factor (vWF) staining

For immunofluorescence staining, ECs were grown on collagen I-coated four-well glass chamber slides to 70–80% confluence before they were fixed for 15 min in 4% paraformaldehyde (Sigma-Aldrich) at room temperature. Fixed ECs were permeabilized with PBS containing 0.1% Triton-X100 (Sigma-Aldrich). Unspecific antibody binding sites were blocked by incubation with 5% donkey serum (Jackson ImmunoResearch Laboratories, Suffolk, UK) for 30 min, then anti-human vWF monoclonal antibody (1:400; clone 2F2A9, BD) was added for at least 30 min. After removal of the unbound antibody and two washing steps in PBS, the cells were counterstained with Cy3-conjugated donkey anti-mouse AffinePure Fab IgG fragments (1:50; Dianova, Hamburg, Germany). Labelled cells were mounted in 75% glycerin containing propylgalate (50 mg/ml) and 4’,6-diamidin-2-phenylindol (DAPI, 200 ng/ml; Roche).

### Mouse xenograft assays

NSG mice were bred and housed at the University Hospital Essen animal care facility. Animal experiments were performed in accordance with institutional guidelines approved by the Animal Care Committee of the University Hospital Essen. 10^5^ UCB-derived CD34^+^ cells were injected intravenously (IV) via tail vein into 8–16 weeks old busulfan pre-conditioned NSG mice. Mice were regularly monitored for any sign of abnormal behavior and sickness. The animals were sacrificed at 8 weeks’ post transplantation for BM isolation from both tibias and femurs of each individual recipient. The bone cavities were flushed with non-supplemented RPMI (Gibco Invitrogen), the BM was collected and erythrocyte lysis was performed. The total white blood cells (WBCs) were stained for flow cytometric analysis with surface markers for assessing the content of human engrafted cells as well as their HSPCs characterization.

### Statistical analysis

Statistical analysis was performed using GraphPad Prism Version 5. All data are given as mean ± standard error of the mean (SEM). Significance analyses for *in vitro* culture was performed with the paired Student t test (*p < 0.05; **p < 0.01; ***p < 0.001). Significance values from the comparison to the Suspension culture are indicated with an asterisk (*), whereas p-values in comparison to AFT024 are labeled with a hashtag (#). Statistical tests for *in vivo* experiments were performed using the Two-way ANOVA.

## Supplementary information

Supplementary Figure
