# Supplementary material for: Human multipotent hematopoietic progenitor cell expansion is neither supported in endothelial and endothelial/mesenchymal co-cultures nor in NSG mice
Source: Sci Rep. 2019 Sep 9;9:12914. doi: 10.1038/s41598-019-49221-x (PMC6733927; doi:10.1038/s41598-019-49221-x)
Supplement: Supplementary file 1 — Supplementary Figure [file 41598_2019_49221_MOESM1_ESM.pdf]

Supplementary Information for

Human multipotent hematopoietic progenitor cell expansion is neither supported in endothelial and endothelial/mesenchymal co-cultures nor in NSG mice

Authors: Stefan Radtke[1;*], André Görgens[1;2;*], Symone Vitoriano da Conceição Castro[1;3], Lambros Kordelas[4], Angela Köninger[5], Jan Dürig[6], Michael Möllmann[6], Peter A. Horn[1], Bernd Giebel[1;#]

List of Supplementary Items:

1. **Figure S1:** Phenotypical characterization of primary human ECs
2. **Figure 2S:** Functional characterization of primary human ECs
3. **Figure S3:** Representative quality control for sort-purified HSPCs
4. **Figure S4:** Expansion of hematopoietic cells in co-culture with ECs and ECs+MSCs
5. **Table S1:** CFC potential of human HSPCs in co-culture with ECs
6. **Table S2:** CFC potential of human HSPCS in co-culture with ECs and MSCs

Figure Legends

Figure S1: Phenotypical characterization of primary human ECs

Analyses of cell surface marker expression on isolated ECs (black histograms) in comparison to isotype-controls (white histogram). Numbers indicate the mean fluorescence intensity (MFI) of the specific staining. HUVEC: human umbilical vein endothelial cells; ECFC: endothelial colony-forming cells.

Figure S2: Functional characterization of primary human ECs

Morphology (Phase), uptake of acetylated low-density lipoprotein (AcLDL, red), intracellular storage of von Willebrand Factor in Weibel-Palade bodies (vWF, green) and formation of tube-like structures in the Matrigel assays of isolated ECs. (Scale-bar: 50µm) HUVEC: human umbilical vein endothelial cells; ECFC: endothelial colony-forming cells.

Figure S3: Representative quality control for sort-purified HSPCs

Representative flow-cytometric assessment of human HSPCs before and after sort-purification for the co-culture with ECs as well as ECs+MSCs.

Figure S4: Expansion of hematopoietic cells in co-culture with ECs and ECs+MSCs

(A) Fold-expansion of differentiated hematopoietic cells lacking CD34 and CD133 ($CD45^{+}$, black) as well as $CD133^{+}CD34^{+}$ (grey) and $CD133^{low}CD34^{+}$ (white) HSPCs in co-culture with human ECs (n=4 for HUVEC 3, all other n=5; mean ± SEM). **(B)** Representative flow-cytometric assessment of MSCs ($CD45^{-}CD90^{+}$) and ECs ($CD45^{-}CD31^{+}$) after 14 days of co-culture with HSPCs. **(C)** Ratio of phenotypical MSCs and ECs after 14 days of co-culture (n=5). **(D)** Fold-expansion of differentiated

hematopoietic cells lacking CD34 and CD133 ($CD45^+$, black) as well as $CD133^+CD34^+$ (grey) and $CD133^{low}CD34^+$ (white) HSPCs in co-culture with human ECs and MSCs (n=5; mean ± SEM).

Figures

Figure S1

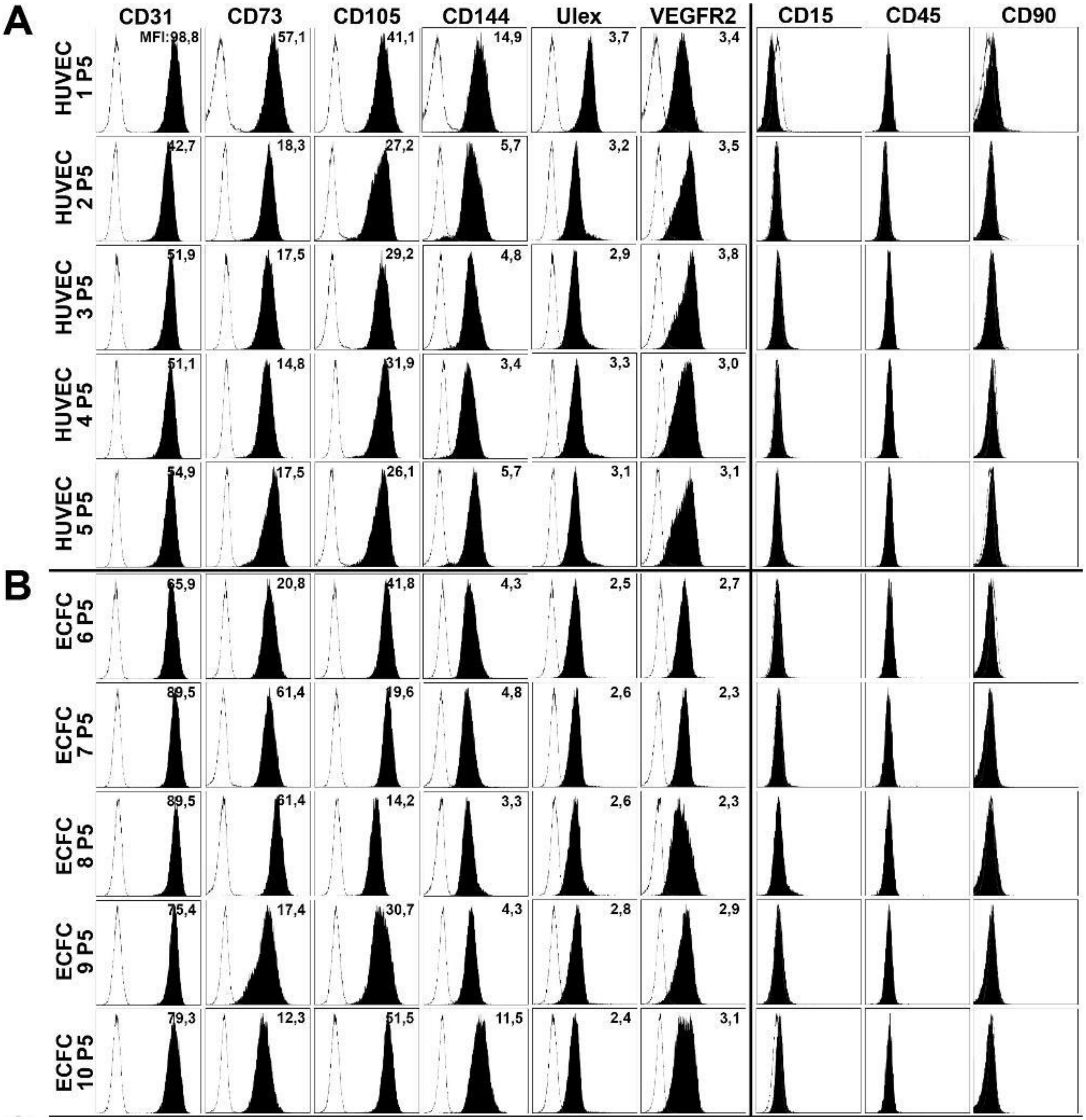

Figure S2

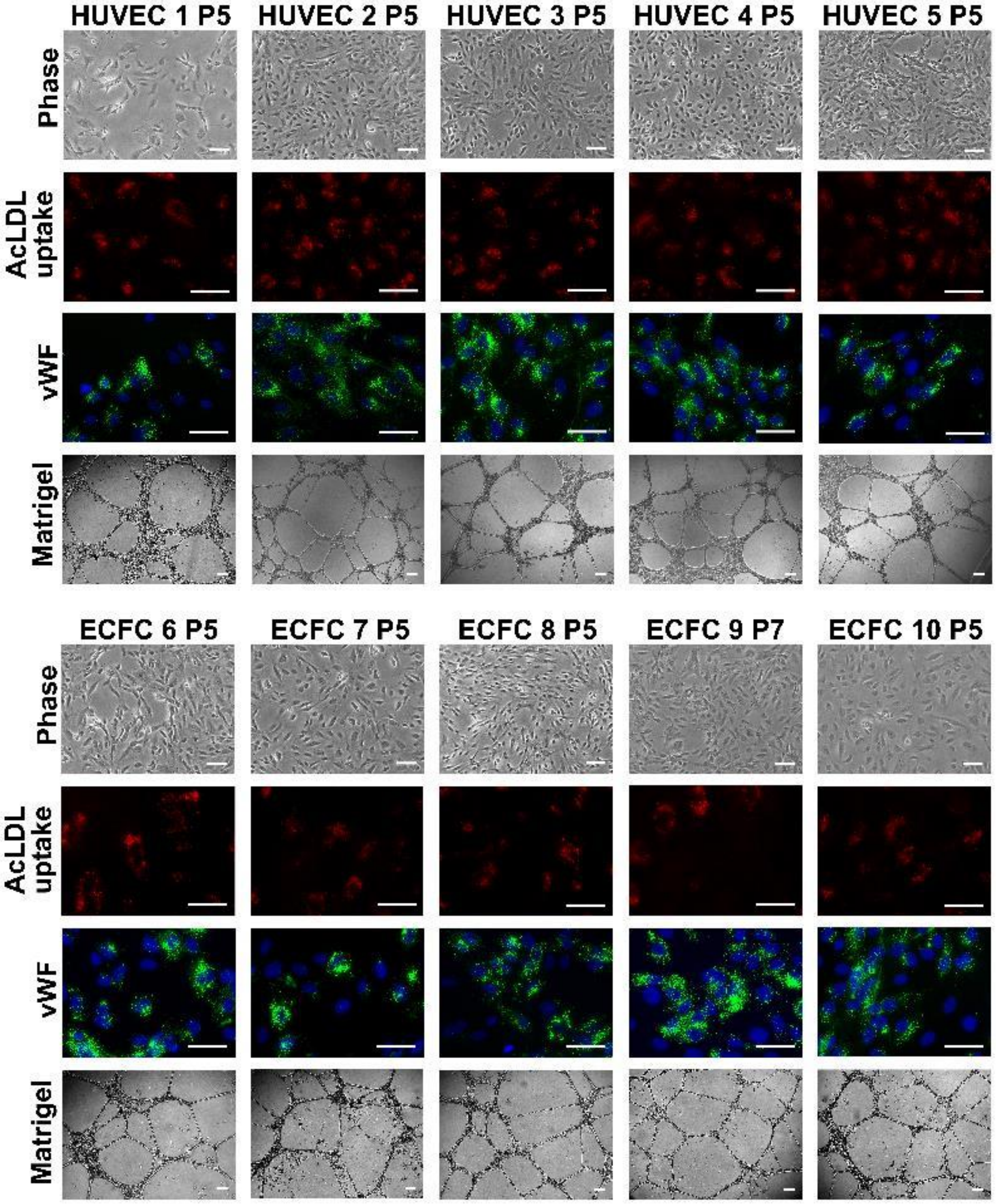
HUVEC 1 P5
HUVEC 2 P5
HUVEC 3 P5
HUVEC 4 P5
HUVEC 5 P5
Phase
AcLDL uptake
vWF
Matrigel
ECFC 6 P5
ECFC 7 P5
ECFC 8 P5
ECFC 9 P7
ECFC 10 P5
Phase
AcLDL uptake
vWF
Matrigel

Figure S3

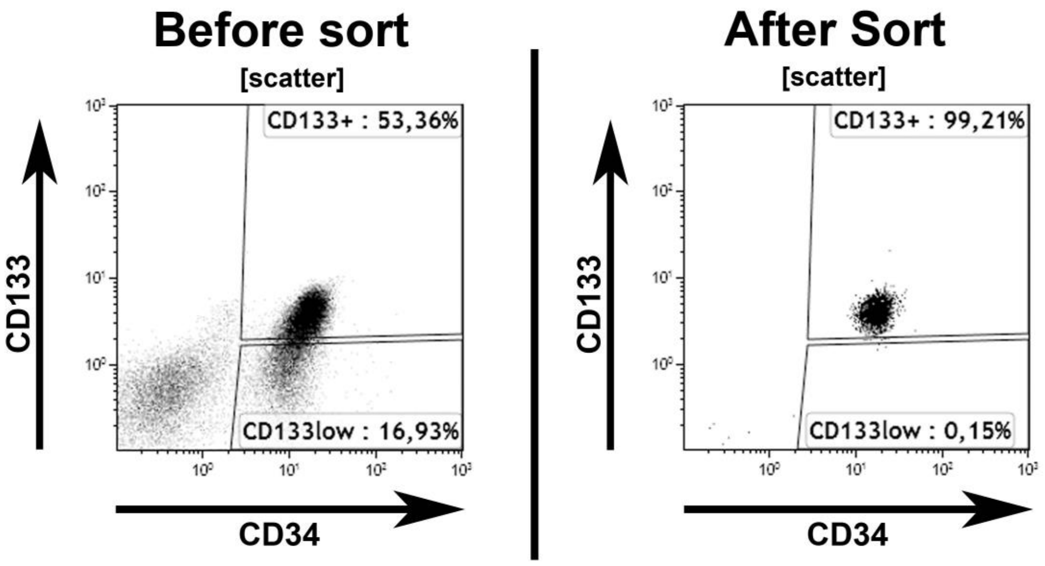
Before sort
[scatter]
CD133+ : 53,36%
CD133low : 16,93%
CD133
CD34
After Sort
[scatter]
CD133+ : 99,21%
CD133low : 0,15%
CD133
CD34

Figure S4

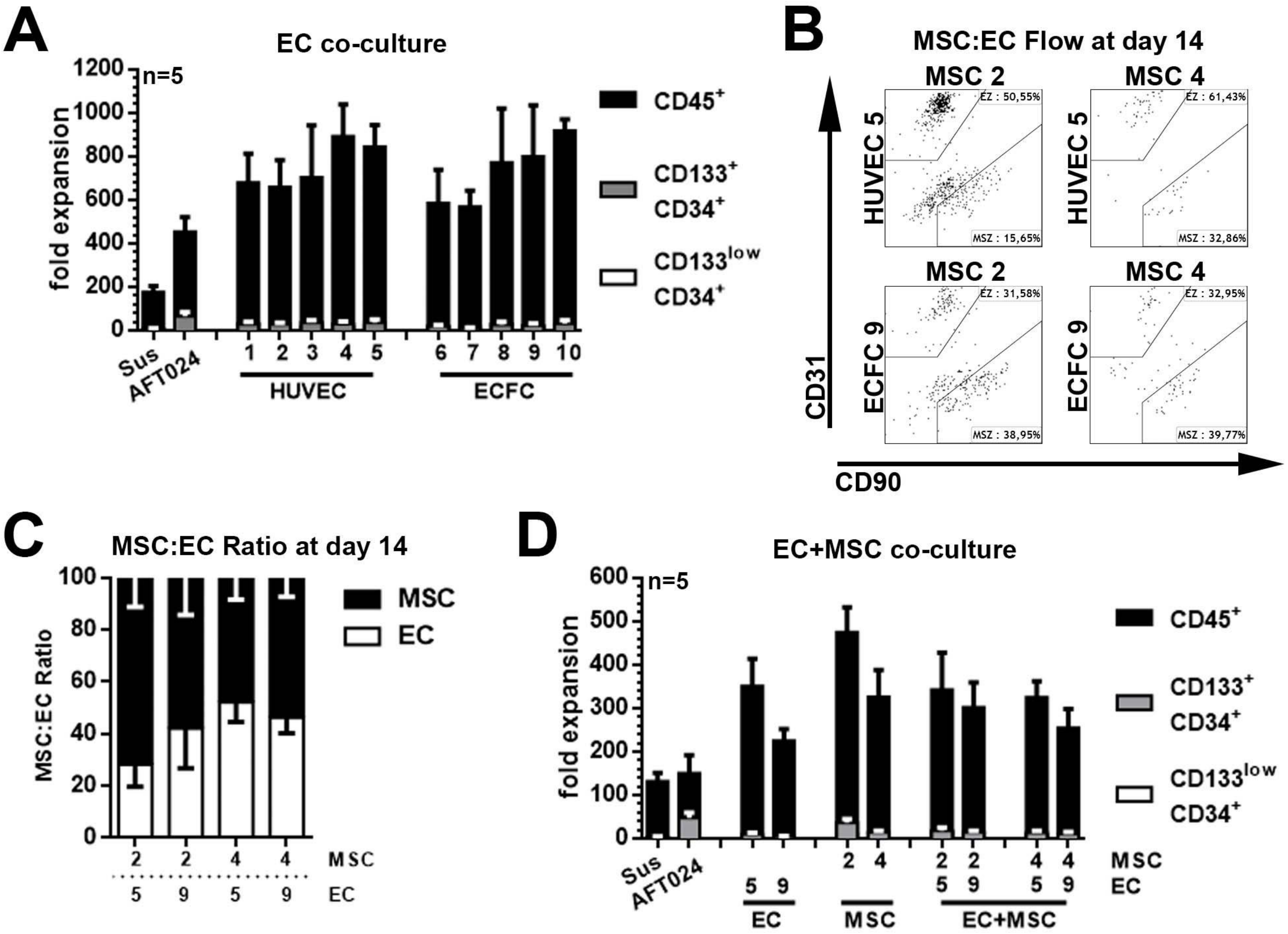
A
EC co-culture
n=5
fold expansion
CD45+
CD133+ CD34+
CD133low CD34+
Sus
AFT024
HUVEC
ECFC
B
MSC:EC Flow at day 14
MSC 2
MSC 4
HUVEC 5
ECFC 9
CD31
CD90
C
MSC:EC Ratio at day 14
MSC:EC Ratio
MSC
EC
D
EC+MSC co-culture
n=5
fold expansion
CD45+
CD133+ CD34+
CD133low CD34+
Sus
AFT024
EC
MSC
EC+MSC

Table S1: CFC potential of human HSPCs in co-culture with ECs

	CD133+						CD133low				
	BFU-E	CFU-M	CFU-G	CFU-GM	CFU-MIX		BFU-E	CFU-M	CFU-G	CFU-GM	CFU-MIX
Suspension	0.00%	2.00%	3.67%	0.00%	0.00%		0.83%	0.17%	2.50%	0.00%	0.33%
	0.00%	1.17%	2.17%	1.33%	0.00%		0.00%	0.17%	4.00%	0.00%	0.00%
	0.00%	2.67%	1.83%	1.00%	0.00%		0.00%	0.33%	1.00%	0.00%	0.00%
	0.00%	4.00%	2.00%	2.00%	0.00%						
AFT024	0.33%	1.00%	5.67%	0.00%	0.00%		0.00%	0.33%	1.33%	0.33%	0.00%
	0.17%	1.17%	1.33%	0.17%	0.00%		0.00%	0.00%	1.33%	0.00%	0.00%
	0.00%	1.33%	2.50%	0.33%	0.00%		0.00%	0.33%	1.67%	0.33%	0.00%
	0.00%	0.67%	2.00%	0.33%	0.00%						
HUVEC 1	0.00%	6.00%	3.00%	0.67%	0.00%		0.00%	0.00%	0.17%	0.00%	0.00%
	0.00%	1.83%	2.17%	1.33%	0.17%		0.17%	0.00%	0.00%	0.00%	0.00%
	0.17%	2.33%	2.00%	2.33%	0.17%		0.00%	0.00%	0.00%	0.00%	0.00%
	0.00%	2.33%	3.33%	2.33%	0.67%						
HUVEC 2	0.00%	5.33%	6.00%	2.00%	0.00%		0.17%	0.00%	1.17%	0.00%	0.00%
	0.00%	3.83%	2.50%	1.00%	0.00%		0.90%	0.00%	0.00%	0.00%	0.00%
	0.00%	6.24%	1.34%	1.56%	0.22%		0.68%	0.00%	0.00%	0.00%	0.00%
	0.67%	4.67%	3.67%	3.33%	0.67%						
HUVEC 3	0.00%	5.67%	7.33%	0.00%	0.00%		0.17%	0.00%	0.17%	0.00%	0.00%
	0.00%	2.67%	2.17%	1.83%	0.17%		0.00%	0.00%	0.25%	0.00%	0.00%
	0.00%	3.31%	6.20%	1.65%	0.21%		0.00%	0.00%	0.33%	0.00%	0.00%
	0.00%	3.33%	3.33%	2.67%	0.00%						
HUVEC 4	0.00%	3.67%	3.00%	0.00%	0.00%		0.00%	0.00%	0.67%	0.00%	0.00%
	0.00%	3.50%	3.17%	1.17%	0.17%		0.00%	0.00%	0.00%	0.00%	0.00%
	0.00%	5.56%	2.22%	1.48%	0.00%		1.16%	0.00%	0.58%	0.00%	0.00%
	0.00%	2.33%	3.67%	5.00%	0.00%						
HUVEC 5	0.00%	7.00%	3.67%	0.67%	0.00%		0.17%	0.00%	0.17%	0.00%	0.00%
	0.00%	3.00%	1.83%	1.17%	0.00%		0.00%	0.00%	0.33%	0.00%	0.00%
	0.00%	2.00%	1.17%	1.33%	0.00%		1.56%	0.00%	0.52%	0.00%	0.00%
	0.00%	2.67%	3.67%	2.67%	0.33%						
ECFC 6	0.00%	1.00%	2.00%	1.00%	0.00%		0.17%	0.00%	0.17%	0.00%	0.00%
	0.00%	3.17%	2.00%	2.33%	0.00%		0.00%	0.00%	0.00%	0.00%	0.00%
	0.00%	3.67%	2.00%	1.83%	0.17%		0.42%	0.00%	0.00%	0.00%	0.00%
	0.00%	3.33%	1.67%	4.00%	0.00%						
ECFC 7	0.00%	2.67%	3.33%	2.00%	0.00%		0.00%	0.00%	0.00%	0.00%	0.17%
	0.00%	1.83%	3.00%	2.33%	0.00%		0.00%	0.55%	0.70%	0.21%	0.03%
	0.00%	6.38%	2.98%	1.70%	0.00%		0.00%	0.00%	0.00%	0.00%	0.00%
	0.00%	3.00%	3.00%	4.67%	0.00%						
ECFC 8	0.00%	2.00%	5.00%	2.00%	0.00%		0.17%	0.00%	0.17%	0.00%	0.00%
	0.00%	4.00%	2.00%	1.33%	0.00%		0.00%	0.00%	0.00%	0.00%	0.00%
	0.00%	3.17%	3.50%	1.67%	0.00%		0.67%	0.00%	0.00%	0.00%	0.00%
	0.00%	3.67%	4.67%	1.33%	0.33%						
ECFC 9	0.00%	7.33%	5.33%	0.33%	0.00%		0.00%	0.17%	0.17%	0.00%	0.00%
	0.00%	2.83%	2.17%	1.50%	0.00%		0.00%	0.00%	0.00%	0.00%	0.00%
	0.00%	3.50%	2.83%	1.17%	0.17%		0.00%	0.00%	0.00%	0.00%	0.00%
	0.00%	3.33%	3.67%	3.00%	0.00%						
ECFC 10	0.00%	3.00%	12.33%	2.33%	0.00%		0.00%	0.17%	0.83%	0.00%	0.00%
	0.00%	1.67%	1.17%	1.00%	0.00%		0.00%	0.00%	0.00%	0.00%	0.00%
	0.00%	3.00%	2.50%	1.67%	0.00%		0.47%	0.00%	0.00%	0.00%	0.00%
	0.00%	3.00%	5.33%	0.33%	0.00%						

Table S2: CFC potential of human HSPCS in co-culture with ECs and MSCs

	CD133+						CD133low				
	BFU-E	CFU-M	CFU-G	CFU-GM	CFU-MIX		BFU-E	CFU-M	CFU-G	CFU-GM	CFU-MIX
Suspension	0.00%	4.41%	8.82%	5.88%	0.00%		1.00%	0.00%	7.00%	0.00%	0.00%
	0.00%	0.00%	16.67%	0.00%	0.00%		0.00%	0.00%	5.88%	0.00%	0.00%
	0.00%	4.05%	6.08%	4.05%	0.00%		0.00%	0.00%	3.51%	0.00%	0.00%
	0.00%	1.50%	4.50%	2.00%	0.00%		0.00%	0.00%	5.00%	0.00%	0.00%
	0.00%	9.86%	10.56%	2.82%	0.70%		0.00%	0.00%	9.00%	0.00%	0.00%
AFT024	0.00%	1.50%	2.00%	0.50%	0.50%		2.00%	0.00%	4.00%	0.00%	0.00%
	0.00%	1.50%	4.00%	0.00%	0.50%		3.50%	0.00%	3.50%	0.00%	1.50%
	0.00%	3.50%	3.50%	0.00%	0.00%		0.00%	0.00%	6.50%	0.00%	0.00%
	0.00%	2.00%	2.00%	0.80%	0.00%		2.45%	0.61%	1.23%	0.00%	0.00%
	1.00%	4.00%	5.00%	2.00%	0.50%		0.00%	0.00%	0.50%	0.00%	0.00%
HUVEC 5	0.00%	1.50%	4.50%	1.00%	0.00%		2.00%	0.00%	4.00%	0.00%	0.00%
	0.00%	2.00%	7.00%	1.00%	0.00%		3.50%	0.00%	3.50%	0.00%	0.00%
	0.00%	1.60%	4.80%	1.60%	0.00%		0.00%	0.50%	3.50%	0.00%	0.00%
	0.00%	4.50%	10.00%	0.00%	0.00%		0.00%	0.00%	0.50%	0.00%	0.00%
	0.00%	8.00%	3.50%	5.00%	0.00%		0.00%	0.00%	2.00%	0.00%	0.00%
ECFC 9	0.00%	1.89%	6.60%	0.00%	0.00%		0.50%	0.00%	3.50%	0.00%	0.00%
	0.00%	1.50%	9.00%	0.00%	0.00%		0.00%	0.00%	1.00%	0.00%	0.00%
	0.00%	0.00%	12.50%	0.00%	0.00%		0.00%	0.00%	1.00%	0.00%	0.00%
	0.00%	4.50%	4.00%	2.50%	0.00%		0.00%	0.00%	2.00%	0.00%	0.00%
	0.50%	5.50%	8.50%	3.00%	0.00%		0.00%	0.00%	4.50%	0.00%	0.00%
MSC 2	0.00%	1.50%	1.50%	0.50%	0.00%		0.00%	0.00%	0.50%	0.00%	0.00%
	0.50%	1.00%	4.50%	0.50%	0.00%		0.00%	0.00%	1.18%	0.00%	0.00%
	0.00%	3.50%	3.50%	1.00%	0.00%		0.00%	0.00%	7.02%	0.00%	0.00%
	0.00%	2.00%	4.00%	0.50%	0.00%		0.00%	0.00%	2.00%	0.00%	0.00%
	0.00%	9.50%	5.00%	2.50%	0.00%		0.00%	0.00%	3.00%	0.00%	0.00%
MSC 4	0.00%	3.00%	5.50%	4.50%	0.00%		0.00%	0.00%	0.00%	0.00%	0.00%
	0.00%	2.00%	6.00%	2.50%	0.00%		0.00%	0.00%	19.05%	0.00%	0.00%
	0.00%	3.50%	4.50%	0.50%	0.00%		0.00%	0.00%	16.67%	0.00%	0.00%
	0.00%	0.50%	4.50%	3.50%	0.00%		0.00%	0.00%	2.50%	0.00%	0.00%
	0.00%	5.50%	3.50%	1.00%	0.00%		0.00%	0.00%	4.00%	0.00%	0.00%
MSC2+ HU5	0.00%	5.50%	3.50%	2.50%	0.00%		0.00%	0.00%	5.00%	0.00%	0.00%
	0.00%	2.50%	5.00%	2.50%	0.00%		0.00%	0.00%	3.70%	0.00%	0.00%
	0.00%	3.03%	4.55%	1.52%	0.00%		0.00%	0.00%	0.00%	0.00%	0.00%
	0.00%	2.50%	8.00%	4.50%	0.00%		0.00%	0.00%	1.00%	0.00%	0.00%
	0.00%	9.50%	7.00%	6.50%	0.50%		0.00%	0.00%	3.00%	0.00%	0.00%
MSC2+ECFC9	0.00%	2.50%	5.00%	0.50%	0.00%		0.00%	0.00%	0.00%	0.00%	0.00%
	0.00%	3.50%	7.50%	2.50%	0.00%		1.09%	0.00%	3.26%	0.00%	0.00%
	0.00%	6.00%	7.50%	6.00%	0.00%		0.50%	0.50%	1.00%	0.00%	0.00%
	0.00%	7.00%	6.50%	4.50%	0.00%		0.00%	0.00%	0.00%	0.00%	0.00%
	0.00%	5.50%	4.50%	3.00%	0.00%		0.00%	0.00%	1.50%	0.00%	0.00%
MSC4+HU5	0.00%	1.50%	3.50%	2.00%	0.00%		0.00%	0.00%	1.00%	0.00%	0.00%
	0.00%	3.50%	5.50%	0.00%	0.00%		0.00%	0.00%	2.50%	0.00%	0.00%
	0.00%	2.20%	7.69%	1.10%	0.00%		0.00%	0.00%	1.00%	0.00%	0.00%
	0.00%	6.50%	5.00%	2.50%	0.00%		0.00%	0.00%	0.00%	0.00%	0.00%
	0.00%	4.00%	6.50%	4.00%	0.00%		0.00%	0.00%	2.00%	0.00%	0.00%
MSC4+ECFC9	0.00%	2.00%	4.50%	2.50%	0.00%		0.00%	0.00%	0.50%	0.00%	0.00%
	0.00%	3.00%	4.00%	1.00%	0.00%		0.00%	0.00%	1.00%	0.00%	0.00%
	0.00%	2.00%	2.50%	2.00%	0.00%		0.00%	0.00%	1.50%	0.00%	0.00%
	0.00%	4.50%	4.00%	2.50%	0.00%		0.00%	0.00%	1.50%	0.00%	0.00%
	0.00%	3.00%	6.50%	4.50%	0.00%		0.00%	0.00%	1.50%	0.00%	0.00%
